# Antler stem cell-conditioned medium stimulates regenerative wound healing in rats

**DOI:** 10.1186/s13287-019-1457-9

**Published:** 2019-11-19

**Authors:** Xiaoli Rong, Wenhui Chu, Haiying Zhang, Yusu Wang, Xiaoyan Qi, Guokun Zhang, Yimin Wang, Chunyi Li

**Affiliations:** 1 Changchun Sci-Tech University, 1699 DongHua St., Shuangyang District, Changchun, Jilin 130022 China; 2 The Scientific Research Center, China-Japan Union Hospital of Jilin University, 126 Xiantai St., Changchun, Jilin 130033 China; 3 Institute of Special Animal and Plant Sciences, Chinese Academy of Agricultural Sciences, 4899 Juye St., Changchun, Jilin 130112 China; 4 School of Life Science, Taizhou University, Taizhou, 318000 China; 5 Key Laboratory of Pathobiology, Ministry of Education, Norman Bethune College of Medicine, Jilin University, 828 Xinmin St., Changchun, Jilin 130021 China; 6 College of Chinese Medicinal Materials, Jilin Agricultural University, 2888 XinCheng St., Changchun, Jilin 130118 China

**Keywords:** Antler stem cells, Conditioned medium, Cell proliferation, Scarless healing, Skin regeneration

## Abstract

**Background:**

When the deer antler is cast, it leaves a cutaneous wound that can achieve scarless healing due to the presence of antler stem cells (ASCs). This provides an opportunity to study regenerative wound healing.

**Methods:**

In this study, we investigated the therapeutic effects and mechanism of antler stem cell-conditioned medium (ASC-CM) on cutaneous wound healing in rats. In vitro, we investigated the effects of the ASC-CM on proliferation of HUVEC and NIH-3T3 cell lines. In vivo, we evaluated the effects of ASC-CM on cutaneous wound healing using full-thickness skin punch-cut wounds in rats.

**Results:**

The results showed that ASC-CM significantly stimulated proliferation of the HUVEC and NIH-3T3 cells in vitro. In vivo, completion of healing of the rat wounds treated with ASC-CM was on day 16 (± 3 days), 9 days (± 2 days) earlier than the control group (DMEM); the area of the wounds treated with ASC-CM was significantly smaller (*p* < 0.05) than the two control groups. Further molecular characterization showed that the ratios of Col3A1/Col1A2, TGF-β3/TGF-β1, MMP1/TIMP1, and MMP3/TIMP1 significantly increased (*p* < 0.01) in the healed tissue in the ASC-CM group.

**Conclusions:**

In conclusion, ASC-CM effectively accelerated the wound closure rate and enhanced the quality of healing, which might be through transforming wound dermal fibroblasts into the fetal counterparts. Therefore, the ASC-CM may have potential to be developed as a novel cell-free therapeutic for scarless wound healing.

## Background

Cutaneous wound healing is the repair of damaged tissue which is designed to restore physiological and anatomical function [1]. The optimal healing of a cutaneous wound requires a well-orchestrated integration of many aspects [2]. Even under optimal conditions, the healing process still leads to fibrosis or a scar. Recently, mesenchymal stem cell (MSC) therapy has been reported as a novel approach in the wound healing field [3, 4]. It is known that MSCs promote wound healing through their differentiation potential, immunomodulatory properties, and paracrine effects [5]. Increasing evidence has shown that MSCs induce wound healing more likely to be through the paracrine pathway [6]. MSCs secrete bioactive molecules to modulate their microenvironment; promote angiogenesis; stimulate the resident cell proliferation; promote cell migration, cell differentiation, survival, and functional recovery of metabolization [7]; and eventually achieve an optimal would healing outcome [8]. This finding offers a potential to develop a cell-free therapeutic for cutaneous wound healing in the clinic. However, identifying and securing suitable sources of stem cells for producing potent bioactive molecules to treat tissue defects and enhance wound healing is one of the main challenges.

Deer antlers are the only known mammalian organ that can completely regenerate once cast from their pedicles, and antler regeneration is a stem cell-based process [9]. A previous study has shown that antler stem cells (ASCs) are unique in that they can not only initiate full regeneration of a mammalian organ, the deer antler, but also promote perfect cutaneous wound healing in a very rapid manner prior to the antler regeneration [10]. In order to evaluate whether this wound healing promotion from the ASCs was species-specific, we injected this cell type into the model rats with cutaneous wounds and found that the wounds achieved similar healing results to those in deers. Therefore, the therapeutic value of ASCs on wound healing is not limited to deers (Rong et al.; in submission). Nonetheless, from the point of view of safety and ethics, ASCs cannot be used directly in the clinical situation, and therefore, an alternative effective approach has to be taken to avoid these problems if the ASCs to be used in clinics.

The aim of this study was to investigate whether the ASC-CM was also effective as a treatment for healing of cutaneous wounds in rats, using both in vitro and in vivo approaches. The ultimate goal of our research was to use the unique antler mode to identify key factors for cutaneous wound healing. In vitro testing is necessary as we wanted to know in pre-hand whether our ASC-CM would likely to work on wound healing before using precious animals. We know wound healing is a complex process, but mainly consisting of two phases: angiogenesis and granulation tissue formation [10]. Endothelial cells are the main cell type for the former, and fibroblasts are the main cell type for the latter. In the present study, therefore, we selected both HUVECs (endothelial cell type) and NIH-3T3 (fibroblast cell type) for in vitro testing to determine whether our ASC-CM could promote proliferation of these two cell types. An improved understanding of the mechanisms of ASC-CM promotion of cutaneous wound healing would undoubtedly contribute to the field of regenerative medicine in general.

## Materials and methods

**Fig. 1:**
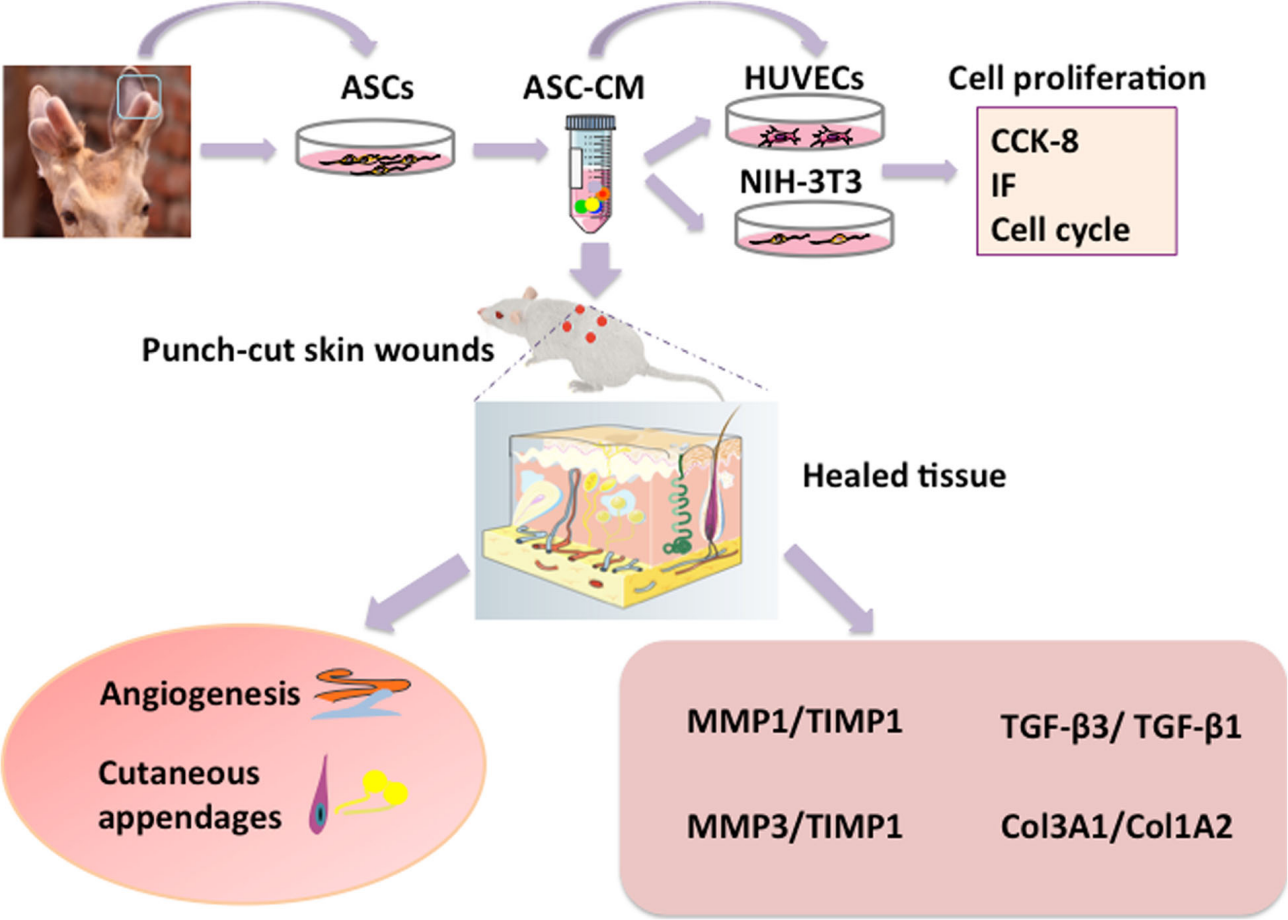
Schematic drawing of experimental procedure**.** ASCs, antler stem cells; ASC-CM, ASC-conditioned medium; IF, immunofluorescence; HUVECs, human umbilical vascular endothelial cells; TGFβ1, transforming growth factor-beta 1; TGF-β3, transforming growth factor-beta 3; MMP1, matrix metalloproteinase 1; TIMP1, tissue inhibitor of metalloproteinase 1; MMP3, matrix metalloproteinase 3

The whole experimental procedure in the study was schematically shown in Fig. 1.

### Isolation and expansion of antler stem cells

Three sticks of antlers were collected from three 2-year-old healthy sika deer (three biological replicates) in accordance with the guidelines of the Animal Ethics Committee of Chinese Academy of Agricultural Sciences. Antler tissues were processed as previously described; briefly, the distal 5 cm of the growing tip was collected, and reserve mesenchyme (RM) layer tissue was cut into 1-mm^3^ pieces for primary cell culture. Detailed procedures for isolation and identification of the primary ASCs have been described previously [11–13]. The isolated ASCs were then cultured in DMEM (Invitrogen, Shanghai, China) supplemented with 10% FBS (Gibco, Australia), 500 U/ml penicillin, and 500 μg/ml streptomycin (Invitrogen, Shanghai, China) at 37 °C with saturated humidity and 5% CO_2_. Facial periosteal cells (FPCs) were obtained following the same procedure reported by Li and Suttie [12].

Human umbilical cord mesenchymal stem cells (hU-MSCs) were isolated from the umbilical cord tissue of a normal cesarean birth according to methods described previously [14]. Briefly, collagenase and hyaluronidase were used to digest the umbilical cord after the outside skin was removed. The isolated hU-MSCs were identified as in our previous paper [15]. Cells were cultured for expansion in DMEM (Invitrogen, Shanghai, China), supplemented with 10% FBS (Gibco, Australia). The cell culture was maintained at 37 °C with saturated humidity and 5% CO_2_. After 48 h, the non-adherent cells were removed by washing, and the medium was changed twice a week. When cells reached 80–90% confluence after 72 h, they were treated with 0.05% trypsin and 0.02% EDTA (Sigma, San Francisco, USA) for 5 min at 37 °C. The first passage cells were washed and harvested by centrifugation at 1000*g* for 5 min, then re-plated at a lower density at 1500 cells/cm^2^.

### Preparation of CMs and optimization of culture parameters

The method for preparation of CMs was as described previously [16]. Briefly, ASCs, hU-MSCs, and FPCs at the third passage were plated in six-well plates at a density of 1 × 10^5^ cells per well. After 48 h, when cells reached approximately 80% confluence, the normal medium (containing 10% FBS) was replaced with serum-free DMEM (Invitrogen, Shanghai, China) after three times of washing with PBS. At 48 h after incubation, the supernatants were harvested as CMs for use in experiments. The designations for these CMs are antler stem cell-conditioned medium (ASC-CM), mesenchymal stem cell-conditioned medium (MSC-CM; positive control), and facial periosteal cell-conditioned medium (FP-CM; positive control) respectively. The DMEM (negative control) and IGF1 (positive control; 5 ng/mL) were subjected to the same cultured conditions as the CMs before using but without presence of cells. The supernatants were collected, pooled, centrifuged at 1000 g, and filtered using 0.22-μm filters. All batches of each type of the collected CMs were pooled together, lyophilized, stored at − 80 °C, and dissolved in DMEM after thawing for the use in in vivo and in vitro studies.

We optimized the culture parameters. Human umbilical vein endothelial cells (HUVECs) were cultured until cells reached 80% confluence, were then plated into 96-well plates at a density of 3000 cells per well, and were incubated for 48 h. We established different ratios of the ASC-CM to the DMEM (containing 10% FBS) of 0:10, 2:8, 4:6, 6:4, 8:2, and 10:0. Cell viability was determined using CCK-8 (Dojindo, Japan), and the corresponding OD value measured at different time points at 490-nm wavelength. The optimal ratio selected for future work was 4:6 (ASC-CM to DMEM).

### Cell proliferation assay

HUVECs and NIH-3T3 cells were plated in 96-well plates until cells reached 80% confluence. Five groups of cells (NC, IGF1, FP-CM, MSC-CM, ASC-CM) were maintained at 37 °C under saturated humidity and 5% CO_2_ and incubated for 5 days. Cell viability was examined by CCK-8 assay daily.

### Cell growth on the CM-coated plates

The 96-well plate was coated with 5 ng/ml of each of the five CMs (5 ng/ml) and dried on the super-clean bench for 2 h. Then, HUVECs and NIH-3T3 cells were plated onto the coating plate at a density of 3000 cells per well, maintained at 37 °C under saturated humidity and 5% CO_2_, incubated for 48 h, and followed using CCK-8 assay.

### Immunofluorescence (IF) staining

HUVECs and NIH-3T3 cells were incubated in 24-well plates for 24 h. Then, the original medium was replaced with one of the five different CMs. At 48 h after incubation, the cells were treated with 4% paraformaldehyde for 10 min and incubated with 1% bovine serum albumin (BSA; Biosharp, Hefei, China) for another 30 min. Then, cells were used to detect the expression of Ki-67: incubated firstly with primary antibody (anti-Ki-67, ab15508, 1:200 dilution, Abcam, Cambridge, UK) for 2 h, and then secondary antibody (anti-rabbit IgG, ab15007, 1:1000 dilution, Abcam, Cambridge, UK) for 1 h. The cell nuclei were labeled with DAPI (Thermal Scientific, Waltham, USA). The intensity was examined by fluorescence microscopy (EVOS, Thermo Scientific, Waltham, USA), and positive cells were analyzed in ten random optical fields.

### Cell cycle analysis

The CMs were added to the HUVEC and NIH-3T3 cultures respectively and incubated for 24 h. Briefly, CM-cultured cells were collected, washed, and suspended in cold 75% ethanol overnight at 4 °C. They were centrifuged, washed, and stained with 50 μg/ml propidium iodide (PI), and 50 μg/ml RNaseA (Beyotime, Shanghai, China) dissolved in 500 μl PBS. The suspension was then incubated for another 30 min and analyzed using flow cytometry.

### Creation of cutaneous wound model in rats and application of treatments

Sprague-Dawley rats (8 weeks old, female, 200 g) were purchased from Liaoning Changsheng Biotechnology Co., Ltd. (Shenyang, China). All experiments were performed in accordance with the guidelines and study protocols of the Animal Experiment Ethic Committee of Chinese Academy of Agricultural Sciences. Briefly, the dorsal area of the rats was shaved under anesthesia. Then four circular holes (8 mm in diameter), full-thickness skin excisional wounds were made on the shaved skin. Skin wounds were created on each side of the midline on rats, with the vertical spacing of 1.8 cm and longitudinal spacing of 2.8 cm from the wound centers. The rats were randomly divided into four groups (*n* = 8/group): DMEM (negative control), EGF (positive control; 5 μg/ml), MSC-CM (positive control; 5 μg/ml), ASC-CM (5 μg/ml). The four treatments were randomly assigned to each of the four wounds. The CM freeze-dried powder was mixed with the hydrogel (Yeasen Biotechnology, Shanghai, China) for each treatment. Then, 200 μl of the ASC-CM hydrogel or the three other control CM hydrogels were pipetted onto the wound every 2 days, and skin damage was recorded photographically every 4 days. The area of each wound was calculated out using Adobe Photoshop CS6. Firstly, lasso tool was used to trace the edge of a wound on a photograph and to circle it, then we calculate the circled area based on the pixels of that area (Additional file 1: Figure S4).

Rats were sacrificed on day 16, and the healing wound tissues collected for later use. The benefits for mixing our CMs with hydrogel before topical application on the cutaneous wounds was that hydrogel can help to seal and moisturize the wounds, slowly release our CMs, prevent wounds from bacterial infection, etc.

### Histological examination

Skin tissue sections were cut at 4-μm thickness and used for staining. After being deparaffinized and rehydrated, hematoxylin and eosin (H&E) staining was performed following the manufacturer’s standardized protocols (Sigma, San Francisco, USA). Immunohistochemistry (IHC) was carried using the Kit (Maixin KIT-9710, Fuzhou, China) following the manufacturer’s instructions. Briefly, the sections were deparaffinized, rehydrated, and incubated in a 99 °C water bath for 15 min, and then 3% H_2_O_2_ was added for 15 min and blocked with 10% normal goat serum for 1 h at 37 °C. Then, sections were incubated with primary antibody anti-PCNA (ab15497, 1:500 dilution, Abcam, Cambridge, UK) and anti-α-SMA (ab5694, 1:500 dilution, Abcam, Cambridge, UK) overnight at 4 °C. Next, the sections were incubated with biotinylated goat-anti-rabbit IgG antibody for 2 h. Diaminobenzidine solution was used as the chromogenic agent for 15 min at 37 °C and sequentially incubated with avidin peroxidase reagent. Hematoxylin was used for counterstaining. Sections were photographed using a microscope (Olympus, Japan).

### Fractionation of CM components

The ASC-CM and the three controls, cytochrome C (internal control, 1 mg/ml), DMEM (negative control) and MSC-CM (positive control) were fractionated, respectively, using an AKTA protein chromatography system (gel filtration column), and ASC-CM. The chromatographic conditions were as follows: SuperdexTM75 10/300 GL gel filtration column, 0.05 mol/L phosphate buffer eluent (pH = 6.9 containing 0.3 mol/L sodium chloride). The flow rate was set to 0.5 ml/min, injection volume was 1 ml, and the UV detection wavelength was 280 nm. Setup procedure: Balance → Correction → Loading → Elution → Balance. In addition, 1.5 column volumes were equilibrated and 2 column volumes were eluted.

### ELISA detection of EGF in the CMs

MSC-CM and ASC-CM were collected respectively after 48 h incubation with serum-free medium. Concentration of epidermal growth factor (EGF) in each type of CM was measured using sandwich ELISA (enzyme-linked immunosorbent assay kits; Minneapolis, MN, USA). After media collection, the cells in each culture well were counted. ELISA values were corrected by using total cell numbers. EGF concentration of each sample was calculated out based on the standard curve.

### RNA extraction and quantitative real-time PCR (qPCR)

We sampled the healed tissue and divided it into four pieces. One was used for RNA extraction with Trizol (Invitrogen, Shanghai, China). The mRNAs were reverse-transcribed into cDNA using an oligo (dT) primer and Superscript II reverse transcriptase (Invitrogen, Shanghai, China). SYBR green dye (Roche, Basel, Switzerland) was used for amplification of cDNA. The levels of Col1A2, Col3A1, transforming growth factor-beta1 (TGF-β1), transforming growth factor-beta3 (TGF-β3), matrix metalloproteinase-1 (MMP1), tissue inhibitor of metalloproteinase 1 (TIMP1), matrix metalloproteinase 3 (MMP3), and the internal standard B2M mRNA were measured with qPCR in triplicate. The primer sequences are listed in Additional file 1: Table S1. All reactions were performed in triplicates, and the data were analyzed using the 2^−ΔΔCt^ method.

### Statistical analysis

Statistical analysis was performed using Prism 6 (Graph Pad software). One-way ANOVA with Dunnett’s multiple comparisons test was used to test for statistically significant differences. All quantitative data were given as the mean ± SD for at least three independent experiments. Differences were considered significant at *p* < 0.05.

## Results

### Determination of the optimal ratio of ASC-CM in culture medium

**Fig. 2:**
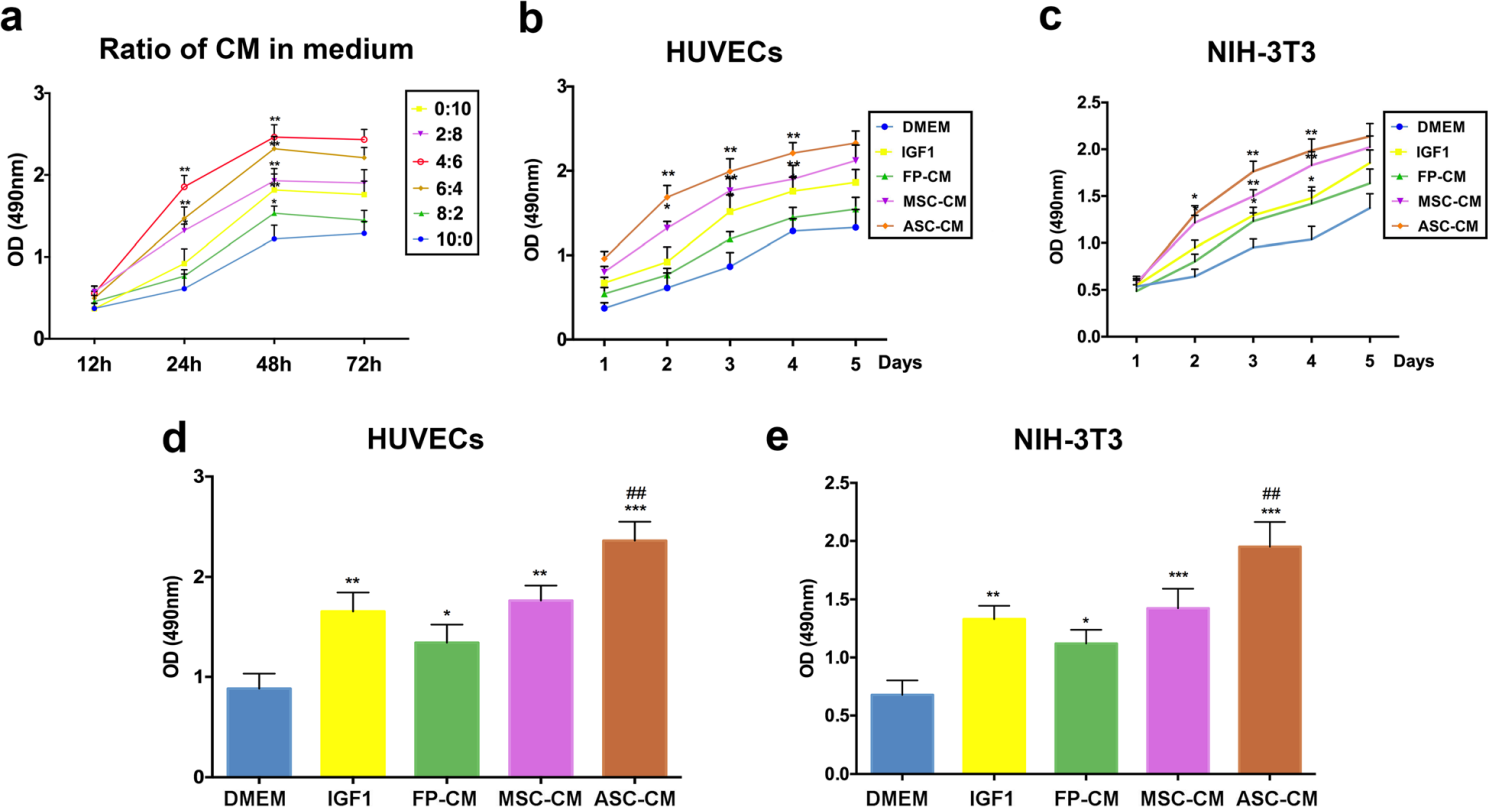
Effects of the ASC-CM on proliferation of HUVECs and NIH-3T3 cells via CCK-8 assay. **a** Optimization of the ratio of ASC-CM to medium using HUVECs (optimal ratio selected was 4:6). **b, c** Effects of ASC-CM in medium (4:6) on proliferation of HUVECs and NIH-3T3. **d, e** Effects of ASC-CM on the coated-surface (4:6) on proliferation of HUVECs and NIH-3T3 at 48 h; HUVECs, human umbilical vascular endothelial cells; FP-CM, facial periosteal cell-conditioned medium; MSC-CM, mesenchymal stem cell-conditioned medium; ASC-CM, antler stem cell-conditioned medium; ^*^p < 0.05, ^**^p < 0.01, ^***^p < 0.001 when compared to 0:10 in **a**, and compared to DMEM in **b**–**e**; ^##^*p* < 0.01 when compared to IGF1; *n* = 3; mean ± SD

The first experiment was set out to determine the optimal ratio of the ASC-CM to DMEM in stimulating HUVEC proliferation (six ratios were included). The results showed that the OD values of three ratios (2:8, 4:6, and 6:4) were significantly higher than 0:10 (i.e., DMEM only; *p* < 0.01). The OD value of the 4:6 ratio at 48 h was the highest (2.31 ± 0.3), and thereafter reached a plateau till 72 h (Fig. 2a). Therefore, the ratio of 4:6 and a culture period of 48 h were selected for the subsequent experiments.

### ASC-CM promoted HUVEC and NIH-3T3 cell proliferation in vitro

Both HUVEC and NIH-3T3 cell types were selected for testing the effects of ASC-CM on wound healing in vitro*,* as wound healing mainly involves two cell types: fibroblasts (main cell type for dermis) and endothelial cells (main cell type for blood vessels).

1. CCK-8 assay either with the ASC-CM in culture medium or with the ASC-CM on coated culture surface

The ASC-CM in culture medium: the highest OD values of ASC-CM were 2.31 ± 0.2 in HUVEC (Fig. 2b, *p* < 0.01) and 2.15 ± 0.3 in NIH-3T3 (Fig. 2c, *p* < 0.01) cell types on day 5, compared to the four control media (DMEM, 0.88 ± 0.15; IGF1, 1.65 ± 0.18; FP-CM, 1.35 ± 0.19 and MSC-CM, 1.76 ± 0.21 in HUVEC; DMEM, 0.67 ± 0.12; IGF1, 1.33 ± 0.14; FP-CM, 1.12 ± 0.11 and MSC-CM, 1.42 ± 0.16 in NIH-3T3).

The ASC-CM being coated on culture surface: results showed the ASC-CM had the strongest effects on proliferation of the HUVEC (OD value, 2.35 ± 0.2; *p* < 0.01) and NIH-3T3 cell types (OD value, 1.96 ± 0.3; *p* < 0.01) at 48 h compared to all four control media (DMEM, 0.88 ± 0.15; IGF1, 1.65 ± 0.18; FP-CM, 1.35 ± 0.19; MSC-CM, 1.76 ± 0.21 in HUVEC; DMEM, 0.67 ± 0.12; IGF1, 1.33 ± 0.14; FP-CM, 1.12 ± 0.11; MSC-CM, 1.42 ± 0.16 in NIH-3T3; Fig. 2d, e).

2) Ki-67 staining

**Fig. 3:**
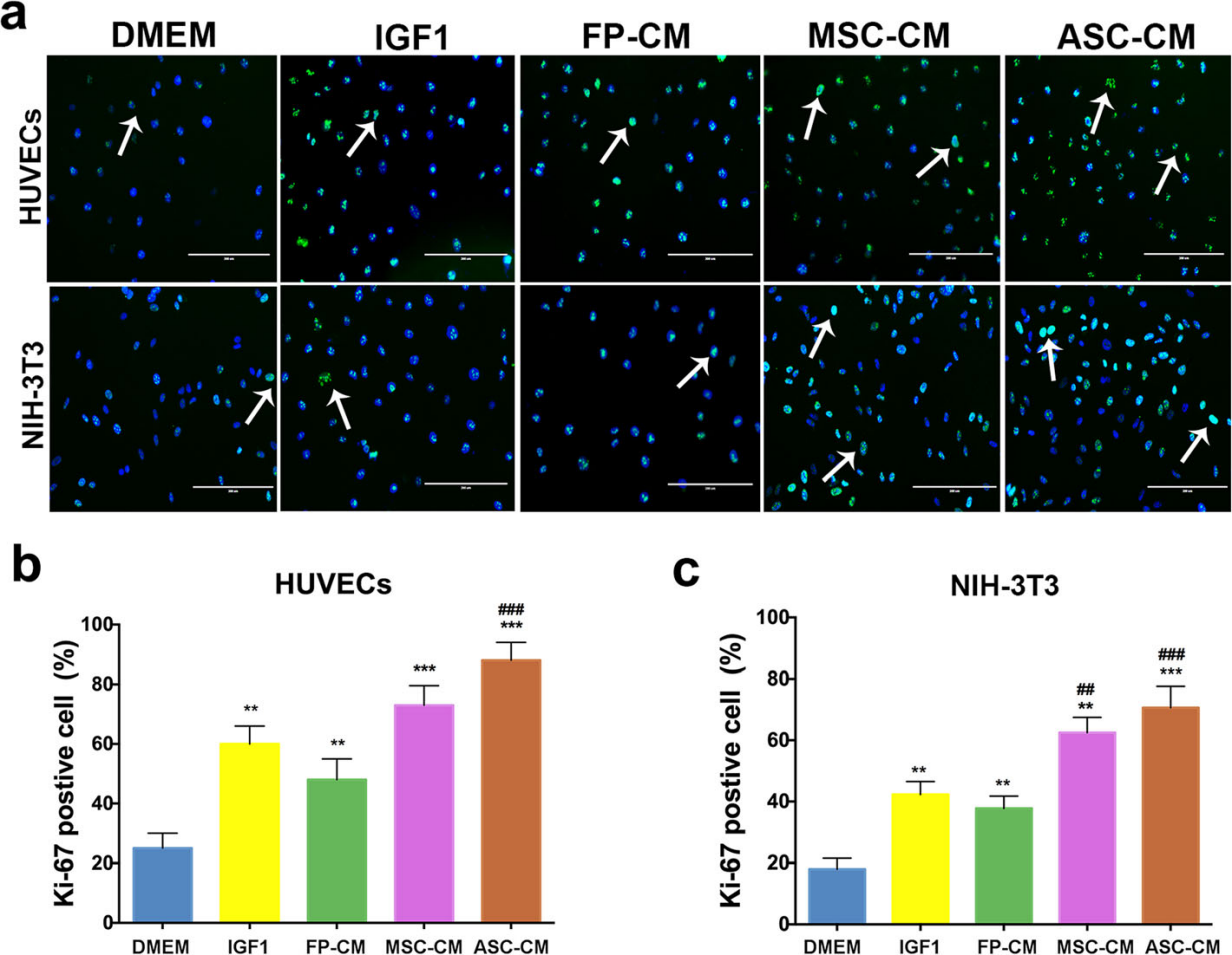
Effects of the ASC-CM on proliferation of HUVECs and NIH-3T3 cells using Ki-67 staining. **a** Immunofluorescent staining of HUVECs and NIH-3T3 using Ki-67 antibody. **b**, **c** Percentages of Ki-67-positive cells in HUVECs and NIH-3T3, respectively. Note that ASC-CM showed the highest percentage of Ki-67-positive cells compared to all four control media in the two cell lines; HUVECs, human umbilical vascular endothelial cells; ^**^p < 0.01, ^***^p < 0.001, when compared to DMEM; ^##^*p* < 0.01, ^###^*p* < 0.001, when compared to IGF1; bar = 200 μm; white arrow, Ki-67-positive cells; *n* = 3; mean ± SD

We found that compared to all four control media, ASC-CM (87.7% and 70.2% respectively) had the highest percentage of Ki-67-positive cells in both HUVEC (DMEM, 24.1%; IGF1, 61.3%; FP-CM, 48.2%; and MSC-CM, 73.5%; *p* < 0.001, Fig. 3a, b) and NIH-3T3 (DMEM, 19.2%; IGF1, 42.6%; FP-CM, 37.8%; and MSC-CM, 62.4%; *p* < 0.001, Fig. 3a, c) cell lines. In addition, the percentage of Ki-67-positive cells in the MSC-CM (62.4%) was significantly higher than that of IGF1 (42.6%; *p* < 0.01) in the NIH-3T3 cells (Fig. 3a, c).

3) Flow cytometry assay

**Fig. 4:**
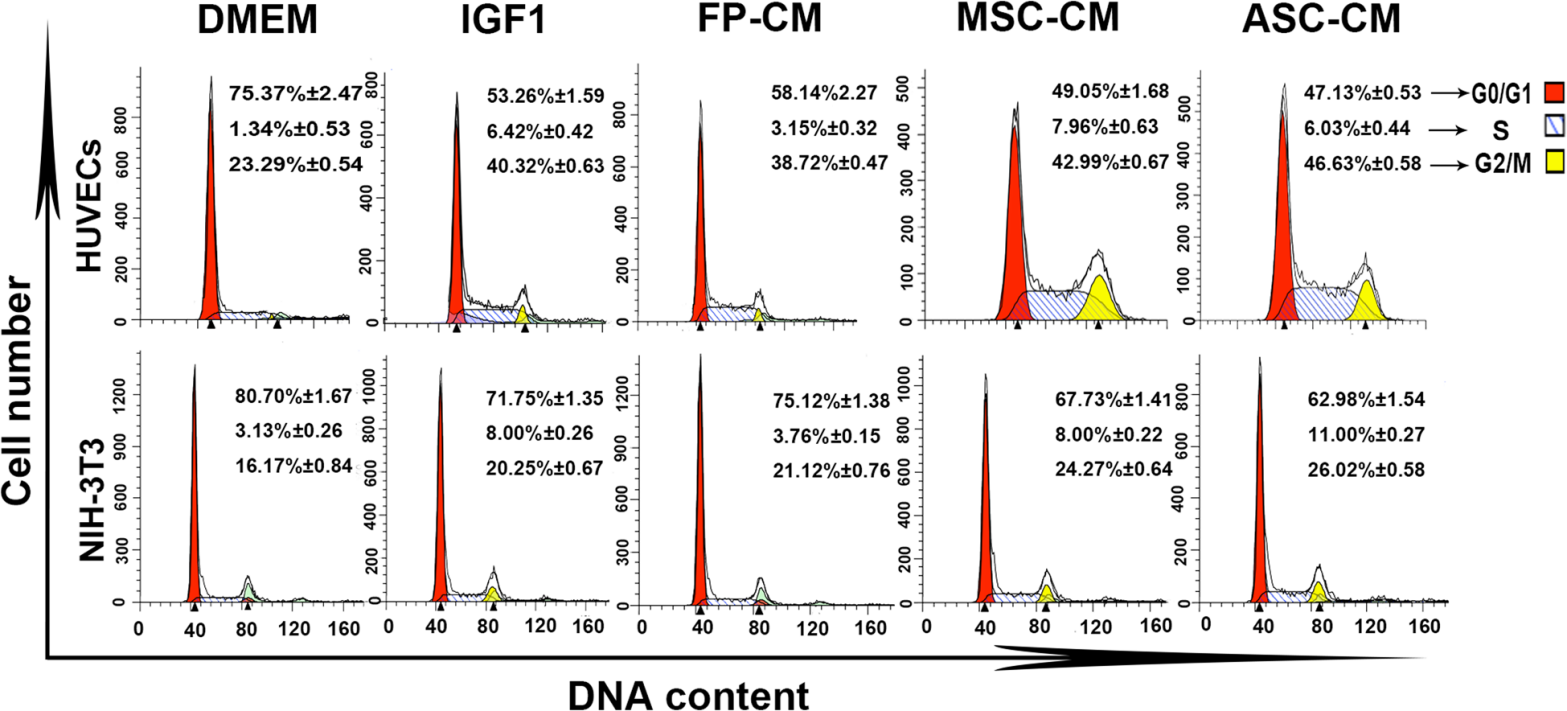
Effects of the ASC-CM on proliferation of HUVECs and NIH-3T3 cells through flow cytometry. Note that ASC-CM had the highest percentage of S+G2/M phase cells compared to the four control media in the two cell lines; HUVECs, human umbilical vascular endothelial cells; *n* = 3; mean ± SD

The results showed that compared to all four control media, the ASC-CM (52.7% and 37.0%, respectively) had the highest percentage of cells at the S+G2/M phase in both HUVEC (DMEM, 24.6%; IGF1, 46.7%; FP-CM, 41.9%; and MSC-CM, 51.0%) and NIH-3T3 (DMEM, 19.3%; IGF1, 28. 3%; FP-CM, 24.9%; and MSC-CM, 32.3%) cell types (Fig. 4). Therefore, the ASC-CM effectively increased percentage of S+G2/M phase cells, which in turn led to enhanced cell proliferation.

Overall, our in vitro results demonstrated that the ASC-CM strongly stimulated proliferation of HUVEC and NIH-3T3 cells compared to the all four control media.

### ASC-CM accelerated wound healing rates in rats

**Fig. 5:**
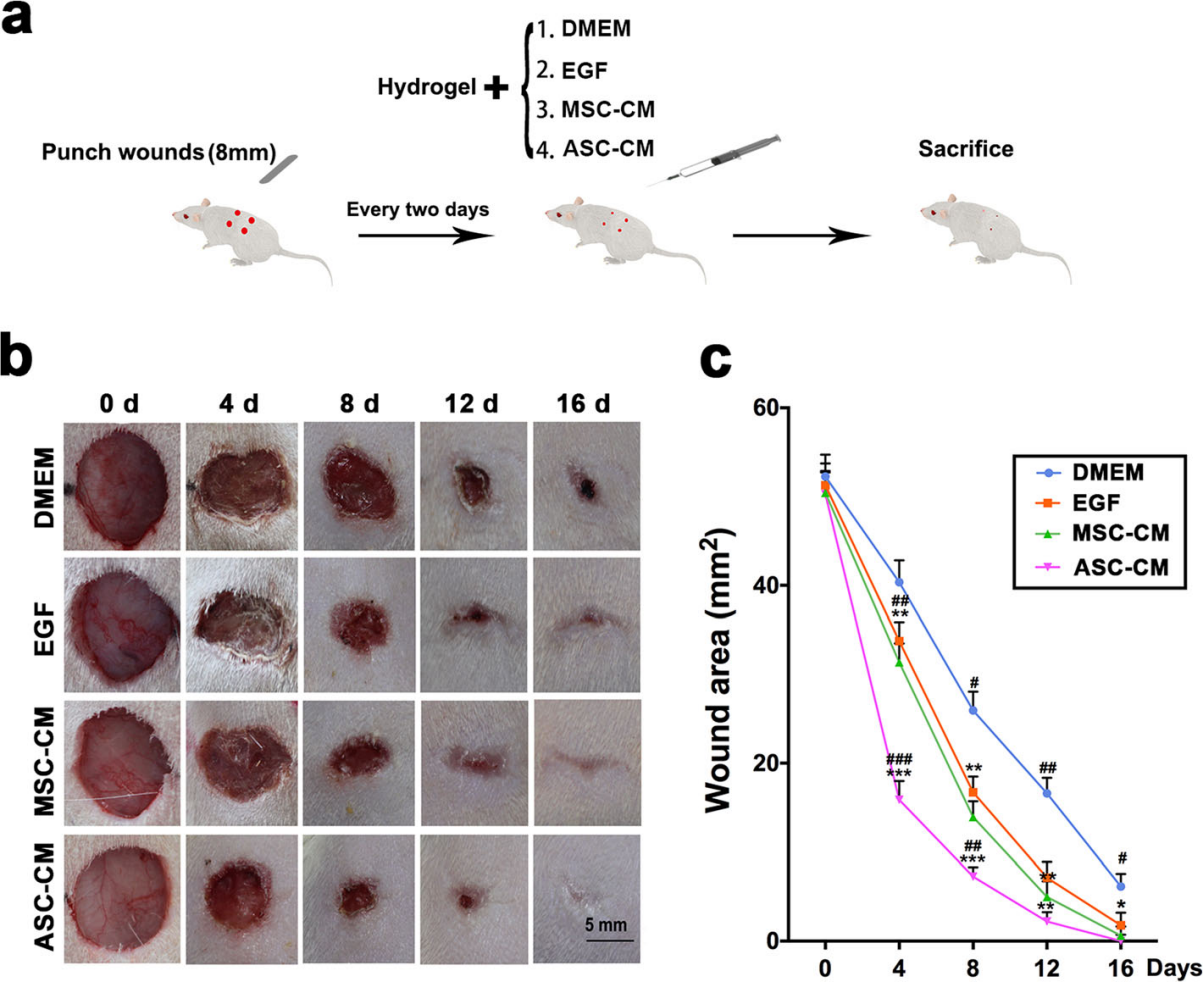
Effects of ASC-CM on rat wound healing rate. **a** Experimental procedure. **b** Overall morphological changes observed during the wound healing. **c** Changes in wound area during healing. Note that the fastest wound healing rate and the smallest wound area occurred in the ASC-CM group compared to other three control groups; MSC-CM, mesenchymal stem cell-conditioned medium; ASC-CM, antler stem cell-conditioned medium; *n* = 8/group; ^*^p < 0.05, ^**^p < 0.01, ^***^p < 0.001 compared to DMEM; ^#^*p* < 0.05, ^##^*p* < 0.01, ^###^*p* < 0.001 compared to EGF; bar = 5 mm; mean ± SD

Full-thickness skin wounds in rats were created and treated with either the ASC-CM hydrogel or the three controls (DMEM hydrogel, EGF hydrogel and MSC-CM hydrogel) respectively (Fig. 5a). The results showed that completion of wound healing morphologically in the ASC-CM treatment group was achieved on day 16, whereas at that time in the three controls with variable sizes of unhealed wounds (DMEM, 6.14 ± 4.1 mm^2^; EGF, 1.79 ± 3.2 mm^2^; MSC-CM, 0.61 ± 2.3 mm^2^, *p* < 0.05; Fig. 5b, c). The fastest healing rate occurred in the ASC-CM group (healing completion achieved on day16 ± 3.5 days; Fig. 5b, c), compared to all three controls (DMEM, 25 ± 1.6 days; EGF, 23 ± 2.6 days; MSC-CM, 20 ± 1.8 days). The wound healing in the ASC-CM group was completed on day 9 (± 2.3 days) and in the MSC-CM group was 5 (± 2.4 days), both were earlier than the DMEM group. These results indicate that ASC-CM has the strongest effect on accelerating wound healing in rats compared to all three control treatments.

### ASC-CM enhanced wound healing quality in rats

**Fig. 6:**
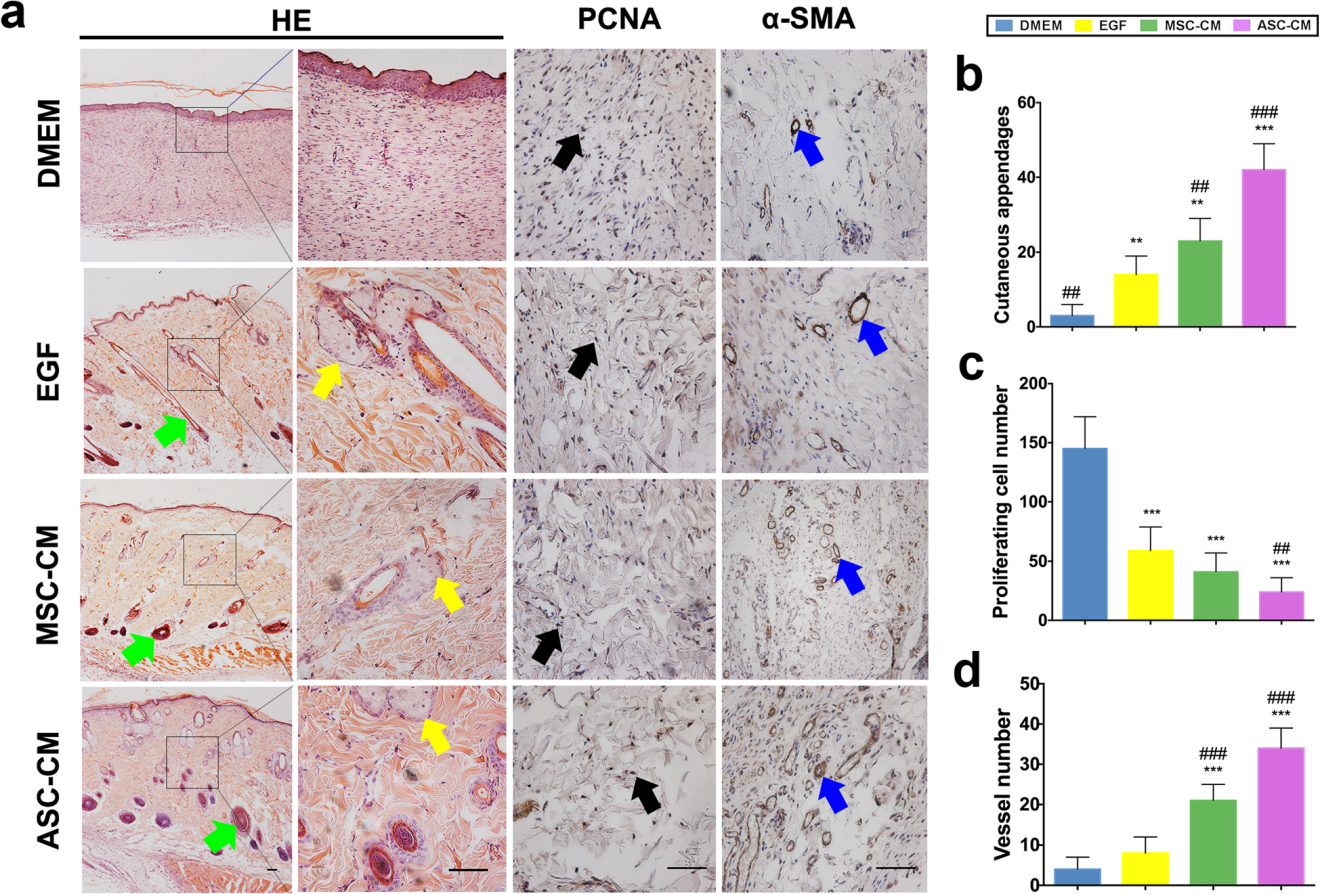
Effects of ASC-CM on rat wound healing quality. **a** H&E staining and IHC evaluation of the wound healing tissues. **b** Number of cutaneous appendages (hair follicles and sebaceous glands)/field (× 40) in the healing tissue. **c** Number of PCNA-positive cells/field (× 100) in the healing tissue. **d** Number of vessels/field (× 100) in the healing tissue. Note that ASC-CM group had the highest number of cutaneous appendages and vessels but the least PCNA-positive cells (on day 16 when wound healing reaching completion) compared to other three types of control groups; green arrow, hair follicle; yellow arrow, sebaceous gland; black arrow, PCNA-positive cells; blue arrow, α-SMA-positive vessels; PCNA, proliferating cell nuclear antigen; α-SMA, α-smooth muscle actin; ^**^p < 0.01, ^***^p < 0.001 compared to DMEM, ^##^*p* < 0.01, ^###^*p* < 0.001 compared to EGF; bar = 1 mm; *n* = 8/group; mean ± SD

Histological examination demonstrated that the ASC-CM treatment had the thickest dermis that contained the highest number of cutaneous appendages (42.3 ± 7.5; including hair follicles and sebaceous glands) compared to all three controls (DMEM, 3.1 ± 2.8; *p* < 0.001; EGF, 17 ± 3.1, *p* < 0.01; MSC-CM, 23.6 ± 6.4, *p* < 0.01; Fig. 6a, b).

The IHC results showed that on day 16 when wound healing had completed in the ASC-CM treatment, this treatment had the least number of the PCNA-positive cells (24.6 ± 12.3) compared to all three controls (DMEM, 145.1 ± 27.4; EGF, 59.3 ± 20.5; MSC-CM, 47.25 ± 18.3; *p* < 0.01, Fig. 6a, c), whereas the vessel number counting results showed that the ASC-CM treatment (34.6 ± 5.3) had the highest vessel numbers compared to all three control treatments (DMEM, 3.9 ± 2.8; EGF, 8.3 ± 4.0; MSC-CM, 21.5 ± 4.1; *p* < 0.001, Fig. 6a, d). These results suggest that the ASC-CM enhanced wound healing in rats may be mainly effected through stimulating regeneration of cutaneous appendages and blood vessels.

### ASC-CM differentially regulated expression of the wound healing related-genes

**Fig. 7:**
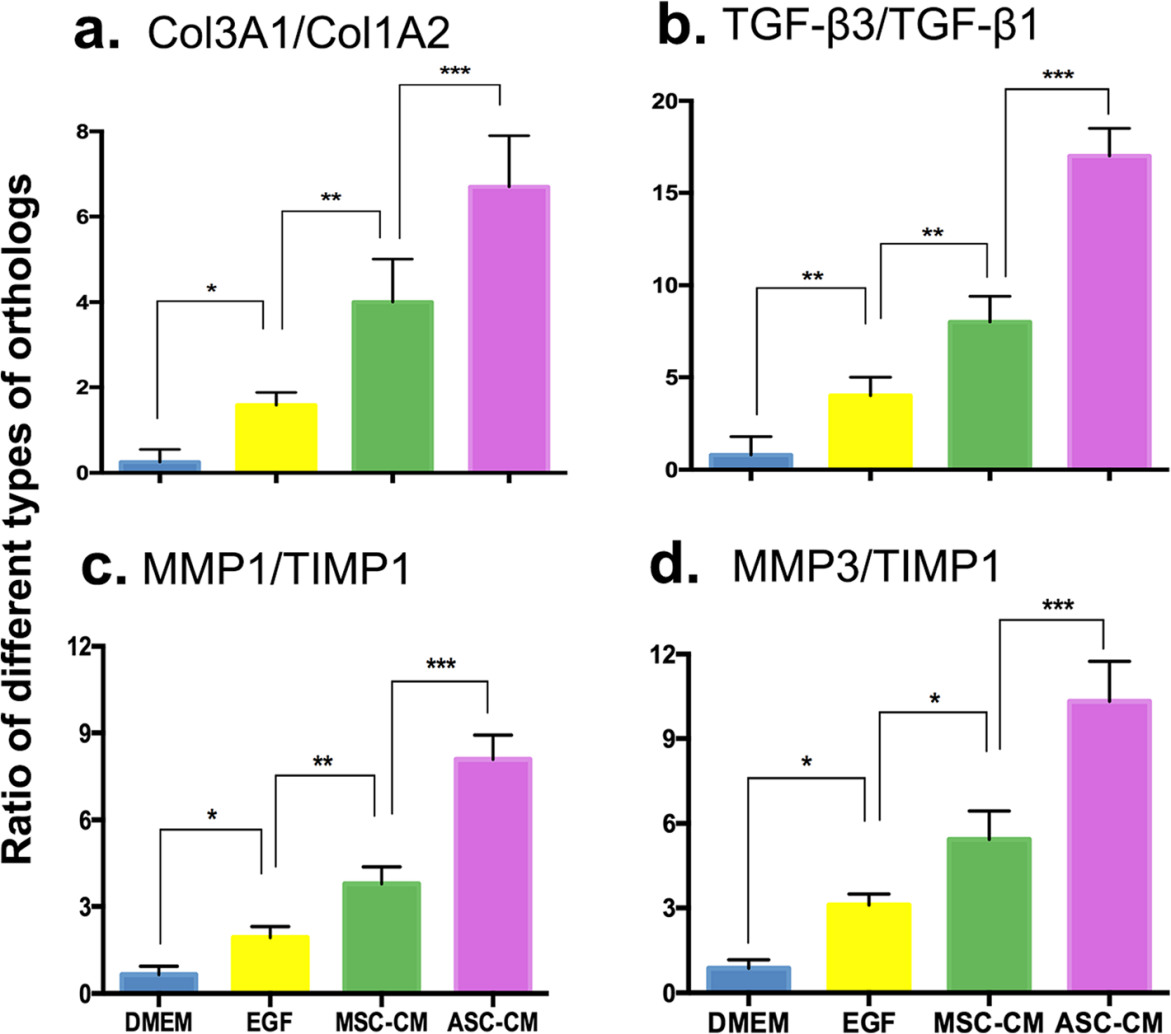
Effects of the ASC-CM on gene expression of the healing tissue via qRT-PCR. **a** Ratio of Col3A1/Col1A2. **b** Ratio of TGF-β3/TGF-β1. **c** Ratio of MMP1/TIMP1. **d** Ratio of MMP3/TIMP1. TGFβ1, transforming growth factor-beta 1; TGF-β3, transforming growth factor-beta 3; MMP1, matrix metalloproteinase 1; TIMP1, tissue inhibitor of metalloproteinase 1; MMP3, matrix metalloproteinase 3; ^*^p < 0.05, ^**^p < 0.01, ^***^p < 0.001; *n* = 3; mean ± SD

The ASC-CM treatment significantly upregulated the expression ratios of both Col3A1/Col1A2 (6.78 ± 1.42, *p* < 0.05; Fig. 7a) and TGF-β3/TGF-β1 (17.32 ± 1.81, *p* < 0.05; Fig. 7b), compared to all three control treatments: Col3A1/Col1A2 (DMEM, 0.25 ± 0.31; EGF, 1.58 ± 0.39; MSC-CM, 4.21 ± 1.32), TGF-β3/TGF-β1 (DMEM, 0.82 ± 0.73; EGF, 4.36 ± 1.52; MSC-CM, 8.24 ± 1.68). Moreover, in the ASC-CM treatment, expression ratios of both MMP1/TIMP1 (8.15 ± 0.94, *p* < 0.05; Fig. 7c) and MMP3/TIMP1 (10.32 ± 1.46, *p* < 0.05; Fig. 7d) were significantly higher than those of the three controls (MMP1/TIMP1: DMEM, 0.65 ± 0.13; EGF, 1.92 ± 0.23; MSC-CM, 3.78 ± 0.45; MMP3/TIMP1: DMEM, 0.85 ± 0.12; EGF, 3.12 ± 0.27; MSC-CM, 5.43 ± 0.48). These results suggest that ASC-CM effectively promote the wound healing likely through activating expression of the genes that recapitulate those in regenerative healing of fetal skin wounds.

### Fractionation and EGF detection of the ASC-CM-soluble components

**Fig. 8:**
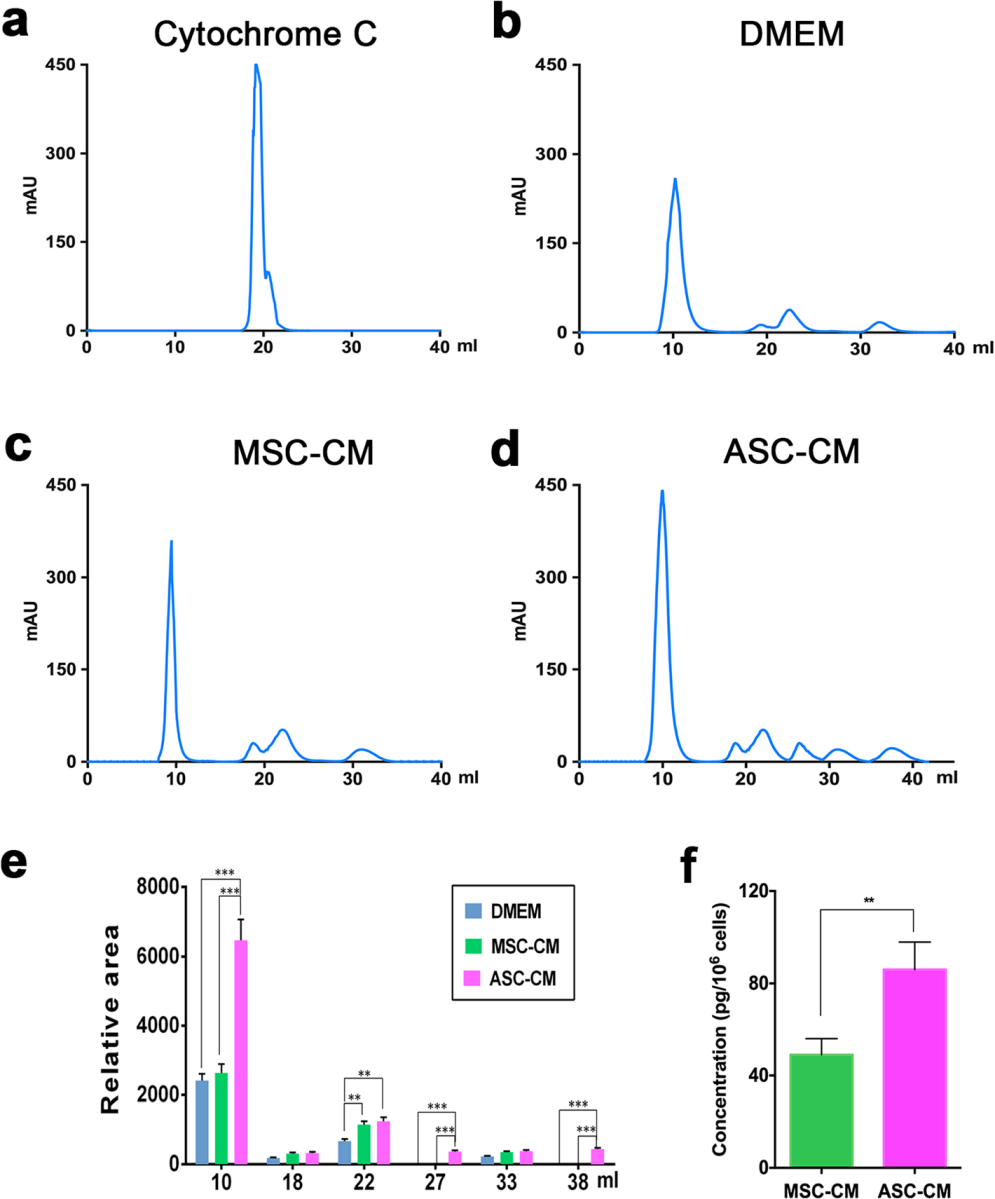
Fractionation of the ASC-CM components using AKTA protein chromatography. **a** Cytochrome C served as an internal reference. **b** DMEM, four peaks identified. **c** MSC-CM, four peaks identified. **d** ASC-CM, six peaks identified. **e** The relative area of peaks in DMEM, MSC-CM, and ASC-CM respectively. Note that the ASC-CM sample had two more peaks appeared at retention volumes of 27 ml and 38 ml than the other two types of media (DMEM and MSC-CM); MSC-CM, mesenchymal stem cell-conditioned medium; ASC-CM, antler stem cell-conditioned medium; each sample in triplicates; **f** EGF concentration in MSC-CM and ASC-CM. Note that EGF in ASC-CM was highly significantly higher than that in MSC-CM (48.73 ± 7.5 and 86.25 ± 12.4 pg/10^6^ cells, respectively, *p* < 0.01). ^**^p < 0.01, ^***^p < 0.001; *n* = 3; mean ± SD

ASC-CM-soluble components were fractionated using protein chromatography. The results showed that relative peak area of the ASC-CM solution (6474 ± 603) was significantly larger (*p* < 0.001) than those of the two control media, DMEM (2410 ± 321) and MSC-CM (2623 ± 251) at retention volumes of 10 ml (Fig. 8b–d). Besides, the ASC-CM had two extra peaks at retention volumes of 27 ml (370 ± 28) and 38 ml (441 ± 43) compared to the two control media (DMEM and MEC-CM). EGF concentrations in ASC-CM and MSC-CM were measured using ELISA kits, and the results showed that EGF in ASC-CM was highly significantly higher than that in MSC-CM (48.73 ± 7.5 and 86.25 ± 12.4 pg/10^6^ cells, respectively, *p* < 0.01). These results suggest that ASC-CM contains more soluble components than MSC-CM, and the better effects of ASC-CM than MSC-CM may stem from higher concentration of growth factors, such as EGF.

## Discussion

To the best of our knowledge, this is the first study to use the ASC-CM to treat cutaneous wounds. We found that the ASC-CM effectively accelerated the rate of wound closure and enhanced healing quality, compared with the previously described conditioned medium for wound healing. The effects of ASC-CM on wound healing may be achieved through transforming the postnatal dermal fibroblasts to fetal fibroblasts, as the genes expressed in the healing tissue of adult wounds recapitulated those of fetal wounds, i.e., the ratios of Col3A1/Col1A2, TGF-β3/TGF-β1, MMP1/TIMP1, and MMP3/TIMP1 resembled those in the fetal healing tissue. Furthermore, in vitro the ASC-CM stimulated proliferation of HUVECs and NIH-3T3 fibroblasts, the two major cell types involved in cutaneous wound healing, suggesting that ASC-CM had at least partially stimulated fibrogenesis and angiogenesis. Therefore, ASC-CM should be a strong candidate to be developed as a potential therapeutic reagent given its apparent superiority to the conditioned media from other types of mesenchymal stem cells (MSCs) that are currently under evaluation for treatment of cutaneous wounds in clinics.

### Why antler stem cells (ASCs)

Stem cell-based therapies have been heralded as a promising new therapy to overcome current limitations in the treatment of cutaneous wounds. The multipotent and self-renewal capacities of MSCs have long been considered advantageous for wound repair via cell replacement, as MSCs have been demonstrated to migrate to sites of injury in response to chemotactic signals in vivo [17]. At the injured sites, MSCs can directly differentiate into various cell types such as keratinocytes, fibrocytes, endothelial cells, and pericytes [18].

Amongst MSCs, ASCs are unique in that they can not only initiate full regeneration of a mammalian appendage (deer antler), but also induce scarless wound healing at the initial stage of antler regeneration [19, 20]. The ability of ASCs to stimulate wound healing over the top of a pedicle stump is also effective on skin elsewhere on the deer’s body, demonstrating that it is not specific to skin type [9]. Recent research has shown that ASCs could stimulate regenerative cutaneous wound healing in rats, and the effects were superior to other tested MSCs from either human or rats, suggesting that the ability of ASCs for healing is not species-specific (Rong et al., in submission). Currently, ASCs have been classified as a special type of MSCs, as they express some of key embryonic stem cell markers, such as Oct4, SOX2, Nanog, TERT, and nucleostemin [21, 22], along with those characteristic of the MSC [23].

### Why ASC-conditioned medium (CM)

The mechanisms by which MSCs ameliorate tissue damage have been debated for many years. Chen et al. [24] summarized two leading theories from the literature as follows: (1) the cell replacement theory (i.e., MSCs physically and functionally substitute for cells lost to tissue damage) and (2) the paracrine signaling theory (i.e., MSCs activate signal transduction pathways in target cells to facilitate tissue regeneration). Nonetheless, more detailed research has found that only a small percentage of grafted MSCs are actually incorporated into, and survive within, lesion tissue, regardless of whether the cells are locally or systemically administered [5]. In some studies, the number of surviving MSCs was found to be too low to explain the significant effects observed following cell administration [24]. Other studies revealed that transplanted MSCs do not necessarily have to be in close proximity to the damaged tissue to promote wound repair, but rather, these cells appear to exert their main therapeutic effects through the secretion of paracrine trophic signals [25].

The proposed action of MSC-CM (containing paracrine factors of MSCs) in enhancing wound healing is via three main pathways [24]: (1) acting as a chemoattractant to recruit specific cell types to the wound site, including epidermal keratinocytes, dermal fibroblasts, endothelial cells, and macrophages [26, 27]; (2) regulating cell migration in response to injury [28]; and (3) stimulating the proliferation of dermal fibroblasts, keratinocytes, and endothelial cells [26, 28–30]. No matter which pathway the MSC-CM may take to effect wound healing, an approach using CM for treating wounds in the clinic could help reduce the biological variability of cell-based therapy, overcoming concerns about cell origin and immuno-compatibility and allowing more precise dosing with purified paracrine components. This approach could facilitate the development of safe and effective cell-free regenerative reagents with predictable and controllable outcomes. In contrast, the ASC approach would need to overcome the ethical and potential xenogeneic cross-infection problems in the transplantation process, compared with MSC transplantation. However, use of ASC-CM treatment of skin wound healing can successfully overcome these two drawbacks.

The fact that paracrine factors from the MSCs play additional roles beyond direct participation of cells in cutaneous wound healing can be better illustrated in the case of ASCs promoting scarless healing during the initial stage of antler regeneration. ASCs reside in the periosteum and do not enter the overlying cutaneous compartment to participate in wound healing [19]. We have shown previously that in the absence of a physical association with the periosteum, however, the wound created following the previous hard antler casting heals as a consequence of ordinary scar. Interposition of an impermeable membrane between the two tissue compartments resulted in wound healing with a scar, whereas a semi-permeable membrane only delayed but did not impede scarless healing [31, 32]. These results further suggest that paracrine factors from the ASCs may be even more potent than those from other types of currently known MSCs, as the ASC factors can traverse up to 1 mm in distance to effect wound healing without requiring the ASCs to enter the healing tissue; in this respect, a distance of 1 mm can be considered as an exceedingly long distance in histological and molecular terms. Besides offering a more efficacious therapy for wounds, the “long-distance” effects of ASC paracrine factors on target tissues would be advantageous when a wound healing reagent is to be developed for clinical use.

### Possible underlying mechanism

The nature of mammalian wound healing in the sense of being scarred or scarless has been attributed to the way that dermal fibroblasts in the healing tissue respond to injury [28]. In this respect, scarless healing in fetal skin is characterized by the absence of contraction and subsequent scarring [33, 34], whereas healing in adult skin involves intense inflammation and scarring [17]. Accumulating evidence demonstrates that fibroblasts from fetal skin and adult skin are different in many aspects in their response to wounds; these include differences in proliferation and migration rates, the ability to form myofibroblasts, ECM synthesis [25], and responses to inflammatory cues [35]. Therefore, dermal fibroblasts of fetal skin are the primary cells involved in regenerative cutaneous healing.

Interestingly, in the initial stage of antler regeneration in an adult mammal, the deer, the process of wound healing over the top of a pedicle stump closely resembles that of fetal skin. In the developing pedicle, there is essentially no contraction detected, as the pedicle skin and the enveloped bone are intimately associated with each other without the interposition by a layer of subcutaneous loose connective tissue. The rate of healing observed in the deer antler is extremely rapid: a wound of 10 cm in diameter can heal within a week, and the nature of the healing is inherently regenerative [19]. At the morphological and histological levels, the initial stage of antler regeneration that is achieved by way of a fetal-like wound healing mechanism including de novo development of skin appendages is well-described [21], but there is very little known at the molecular level. However, recent research has found that deletion of the pedicle periosteum (within which ASCs reside) completely abrogated regenerative healing and resulted in a scar formation [36], suggesting that the pedicle periosteum played a pivotal role in this regenerative wound healing. Consequently, we believe that ASCs have likely converted the phenotypes of adult fibroblasts in the healing dermis into fetal-like phenotypes through paracrine influences which have facilitated the fetal-like wound healing.

Fetal fibroblasts differ from adult fibroblasts in collagen synthesis in terms of speed of deposition, variation in collagen type ratios, and quantity of collagen. Most striking is the persistence of excess Col3 over Col1, with healed wounds in the fetus showing ratios of Col3/Col1 that remain at around 3:1 instead of the 1:3 ratio observed in adult healed wounds [37]. Chen et al. [24] have shown that higher levels of Col3 yield smaller, reticular structures with more cross-linking than Col1 and contributed toward scarless wound healing. In the present study, we found that the ratio of Col3A1/Col1A2 in healed tissue in the ASC-CM treatment group could match that of fetal skin (see Fig. 7a, b). The TGF-β family has been shown to be closely involved in scar formation and that TGF-β3 (so-called anti-fibrotic isoform) is more abundant than TGF-β1 (so-called fibrotic isoform) in fetal wounds [38]. In fetal fibroblasts, the levels of TGF-β1 were downregulated, whereas the levels of TGF-β3 were upregulated [39, 40]. TGF-β1 stimulates fibroblasts to form myofibroblasts, which are the major players in wound contraction [24]. In the present study, we found that expression of TGF-β1 in the healed tissue was downregulated, whereas TGF-β3 was upregulated, which makes the ratio of TGF-β3/TGF-β1 closely resembles that of fetal skin (see Fig. 7c, d).

ECM remodeling is another important component of scarless wound healing in fetal tissue. It requires the coordinated regulation of MMPs and their inhibitors, the TIMPs [40]. Scarred wound healing has been found to be correlated with low MMP activity and high TIMP activity. In the present study, we found that the ratios of MMP1/TIMP1 and of MMP2/TIMP1 increased in the healed tissue following the treatment with ASC-CM. Therefore, remodeling of the ECM structure in the healing tissue must have been promoted in the ASC-CM group thus constituting a microenvironment that is permissive for cell migration and proliferation in wound healing. All of the above findings demonstrate that dermal fibroblasts in the healing tissue represent a phenotype similar to that of fetal fibroblasts after application of ASC-CM. Therefore, treatment methods that can modify the phenotype of adult fibroblasts into fetal-like fibroblasts may provide a promising means through which the quality of wound healing can be improved, even to achieve a scarless wound healing.

Compared to acute wounds that were used as a model system in the present study, chronic wounds in people have been sharply increased in recent years due to an ever-aging population, increasing number of obese and diabetic patients, and cardiovascular disease [41]. Therefore, more effective treatments for chronic wounds are urgently needed to curb this increase. Therapy with MSCs has been found to be attractive due to their differentiating potential, their immunomodulating properties, and their paracrine effects [42]. It is envisaged that ASC-CM should have a better outcome when treating chronic wounds compared to MSC-CM, as the ASCs were more effective on treating acute wounds (present study) and have more potent paracrine action [31].

## Conclusions

In conclusion, ASC-CM was effective on treating full-thickness skin wounds in rats, which might be through transforming wound dermal fibroblasts into the fetal counterparts. Overall, ASC-CM has great potential to be developed as a novel cell-free therapeutic approach for cutaneous wound healing in general.

## Supplementary information

**Additional file 1: Figure S1.** Morphological observation and colony formation of ASCs. **Figure S2.** Expression of surface stem cell markers in ASCs. **Figure S3.** Multipotency of ASCs. **Figure S4.** The area of each wound was calculated out using Adobe Photoshop CS6. **Table S1.** Primers used for qRT-PCR.

## Data Availability

The datasets used and/or analyzed during the present study are available from the corresponding author on reasonable request.

## Abbreviations

ASC-CM: Antler stem cell-conditioned medium
ASCs: Antler stem cells
BSA: Bovine serum albumin
FP-CM: Facial periosteal cell-conditioned medium
FPCs: Facial periosteal cells
H&E: Hematoxylin and eosin
hU-MSCs: Human umbilical cord mesenchymal stem cells
HUVECs: Human umbilical vascular endothelial cells
IF: Immunofluorescence
IGF1: Insulin like growth factor 1
IHC: Immunohistochemistry
MMP1: Matrix metalloproteinase 1
MMP3: Matrix metalloproteinase 3
MSC: Mesenchymal stem cell
MSC-CM: Mesenchymal stem cell-conditioned medium
PCNA: Proliferating cell nuclear antigen
PI: Propidium iodide
qPCR: Quantitative real-time PCR
RM: Reserve mesenchyme
TGFβ1: Transforming growth factor-beta 1
TGF-β3: Transforming growth factor-beta 3
TIMP1: Tissue inhibitor of metalloproteinase 1
α-SMA: α-Smooth muscle actin
