# Supplementary material for: Antler stem cell-conditioned medium stimulates regenerative wound healing in rats
Source: Stem Cell Res Ther. 2019 Nov 19;10:326. doi: 10.1186/s13287-019-1457-9 (PMC6862758; doi:10.1186/s13287-019-1457-9)
Supplement: Supplementary file 1 — Additional file 1: Figure S1. Morphological observation and colony formation of ASCs. Figure S2. Expression of surface stem cell markers in ASCs. Figure S3. Multipotency of ASCs. Figure S4. The area of each wound was calculated out using Adobe Photoshop CS6. Table S1. Primers used for qRT-PCR. [file 13287_2019_1457_MOESM1_ESM.docx]

**Supplemental Materials**

**
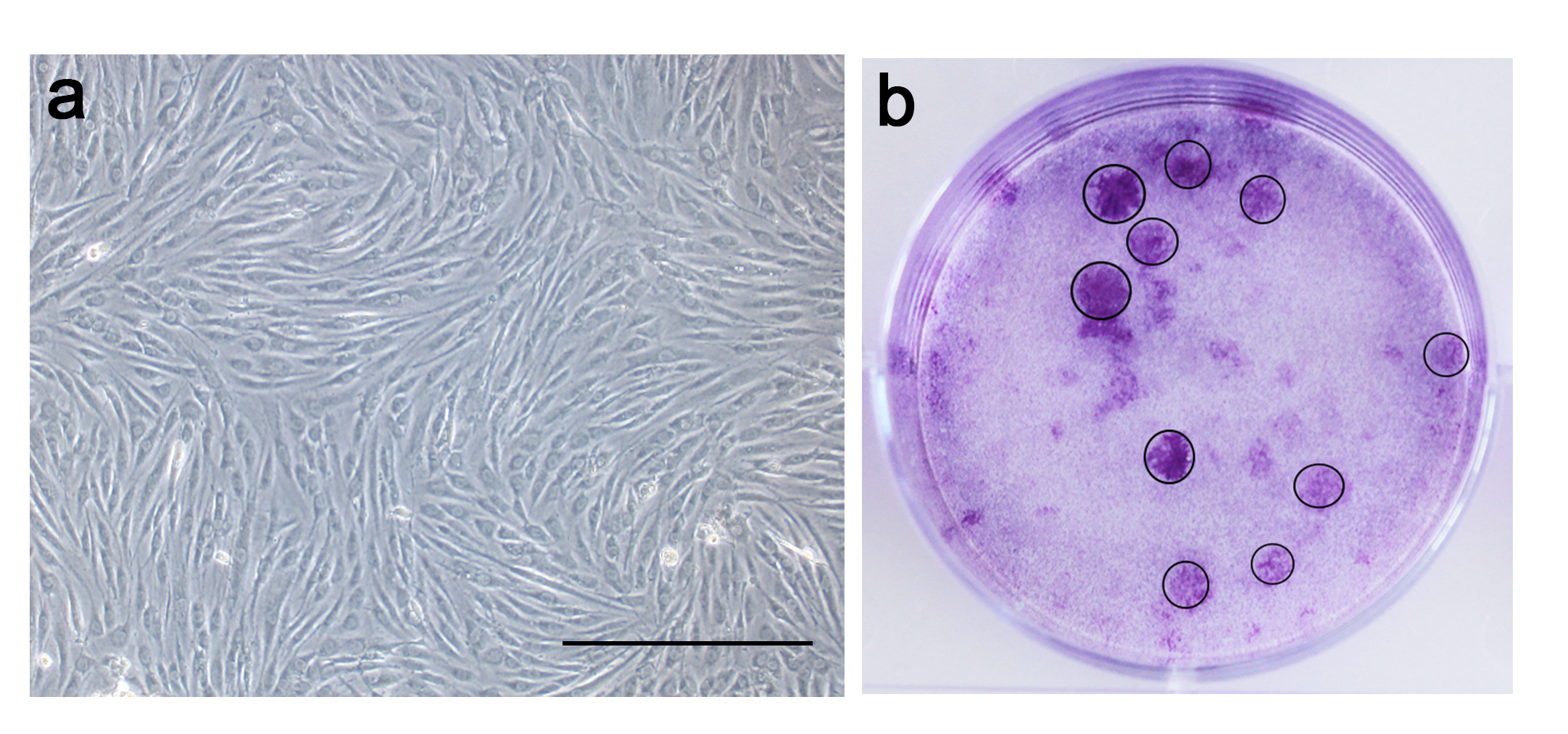
**

**Fig. S1** Morphological observation and colony formation of ASCs. **a** Cells from antler reserve mesenchyme (RM) layer tissues were isolated and cultured as described in methodology section; their morphology was monitored under microscope (Passages≤5); Bar = 100 μm. **b** Colonies formed after seeding 100 cells/ well at day 14, and were stained with crystal violet dye; Bar = 5 mm; ASC, antler stem cell; RM, reserve mesenchymal (growth center of antler containing the stem cells).


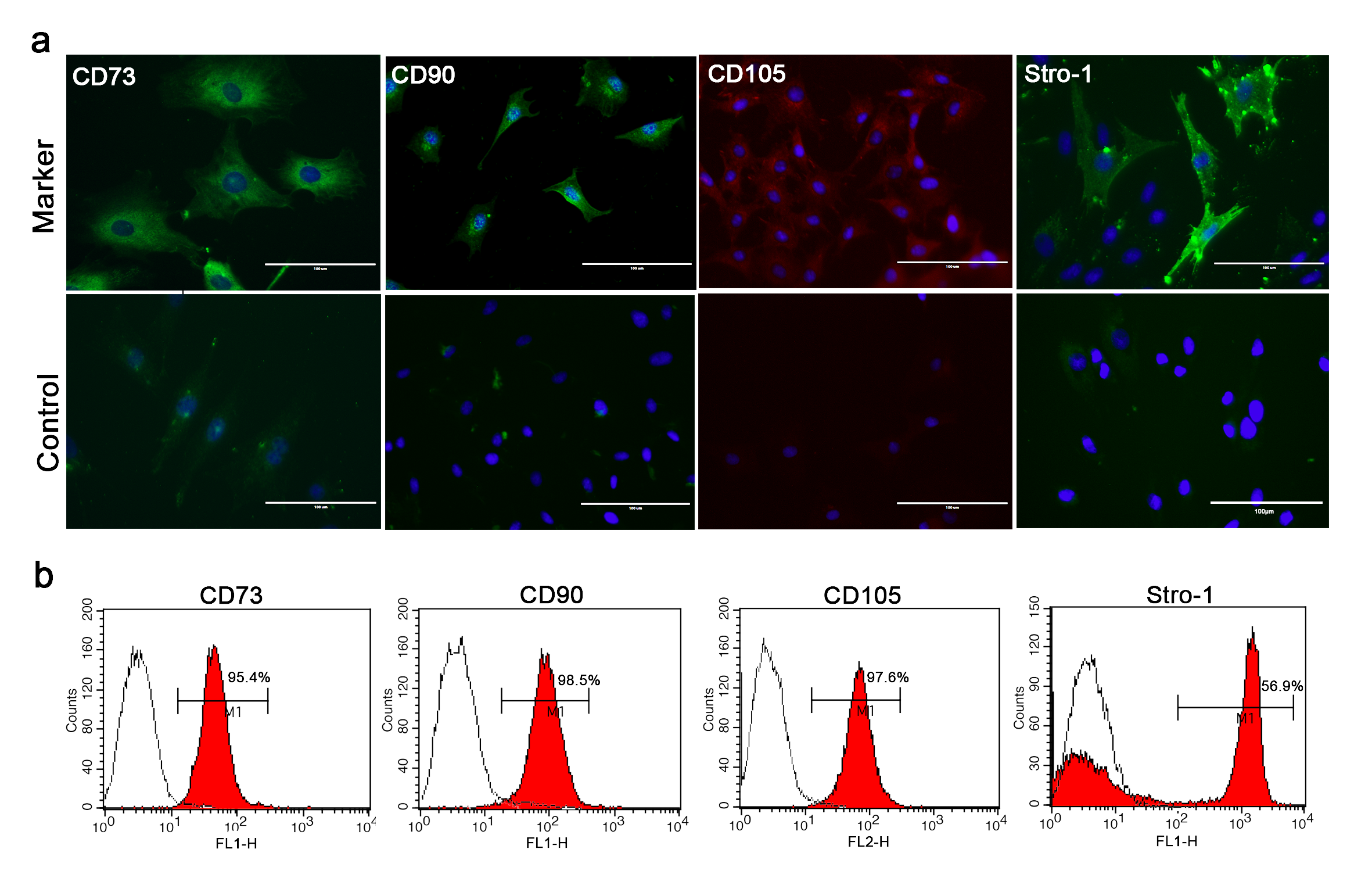


**Fig. S2**  Expression of surface stem cell markers in ASCs. **a** Immunofluorescence staining of ASCs. Classical surface markers of stem cells (CD73, CD90, CD105, Stro-1) were detected using immunofluorescence staining (Green or red). Cell nuclei were counterstained with DAPI (Blue). Bar = 100 μm. **b** Flow cytometry analysis of ASCs. Expressions of indicated antigen (CD73, CD90, CD105, Stro-1) are shown in red histograms in contrast to isotype controls (black histograms). Values showed positive expression patterns of the indicated antigen.


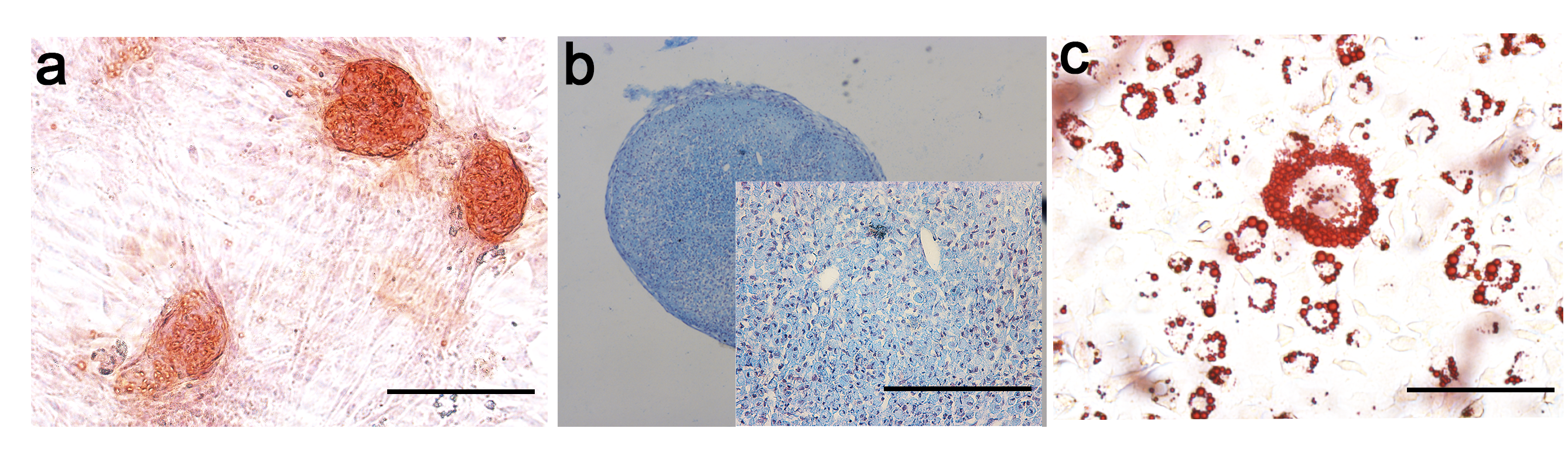


**Fig. S3** Multipotency of ASCs. **a** Osteogenic differentiation; differentiated cells stained strongly with Alizarin Red S. **b** Chondrogenic differentiation; the stained tissue displayed a typical cartilaginous tissue phenotype. **c** Adipogenic differentiation; ASCs exhibited Oil Red-O positive lipid droplets. Bar = 100 μm.


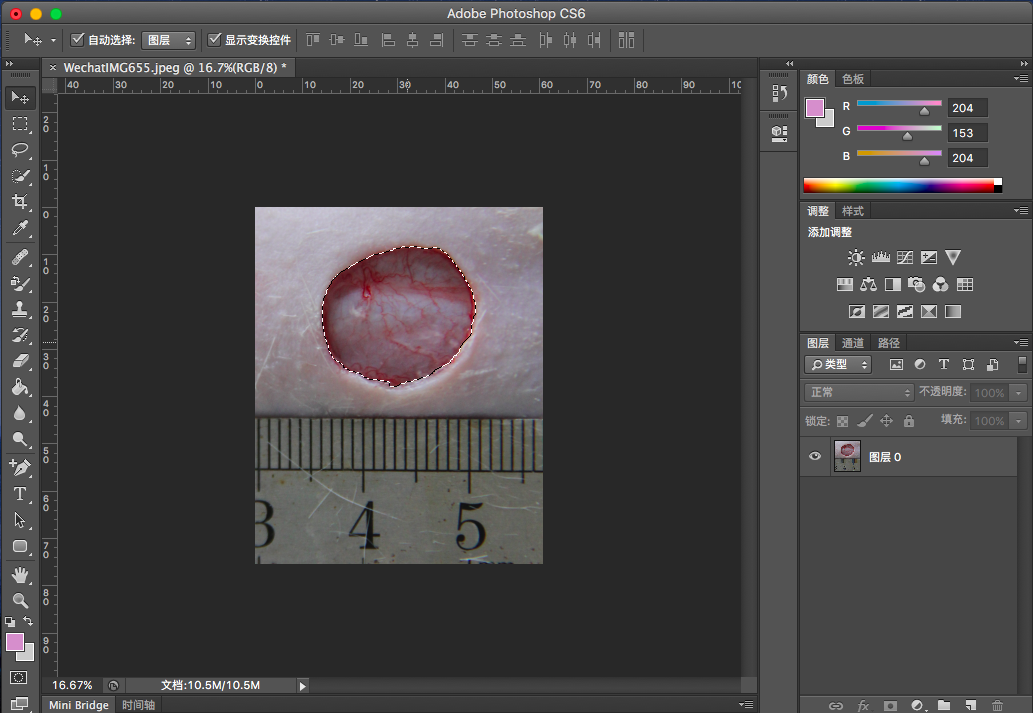


**Fig. S4** The area of each wound was calculated out using Adobe Photoshop CS6. Firstly, lasso tool was used to trace the edge of a wound on a photo and to circle it, then calculate the circled area based on the pixels of that area.

**Table S1. Primers used for** **qRT-PCR**

| Gene name | Primers | Sequences | Product size (bp) |
| --- | --- | --- | --- |
| B2M | Forward | gctccttttgtggctgga | 151 |
|  | Reverse | aagacgccaggtttgctg |  |
| Col1A2 | Forward | ggtgcccctggagagaat | 158 |
|  | Reverse | ggaccagcagacccaatg |  |
| Col3A1 | Forward | gtccacgaggtgacaaaggt | 189 |
|  | Reverse | catcttttccaggaggtcca |  |
| TGFβ1 | Forward | atacgcctgagtggctgtct | 153 |
|  | Reverse | tgggactgatcccattgatt |  |
| TGFβ3 | Forward | ctctctgtccacttgcacca | 185 |
|  | Reverse | tgcatctcttccagcaactcc |  |
| MMP1 | Forward | gctttggcttccctagcagtg | 201 |
|  | Reverse | tcgcctttttggaaaacatc |  |
| MMP3 | Forward | ccaccgagctatccactcat | 159 |
|  | Reverse | gtccggtttcagcatgtttt |  |
| TMIP1 | Forward | catggagagcctctgtggat | 210 |
|  | Reverse | atggctgaacagggaaacac |  |
